# Antibacterial Effect of Silver Nanoparticles Against Four Foodborne Pathogens

**DOI:** 10.5812/jjm.8720

**Published:** 2014-01-01

**Authors:** Mehdi Zarei, Amirhesam Jamnejad, Elahe Khajehali

**Affiliations:** 1 Department of Food Hygiene, Faculty of Veterinary Medicine, Shahid Chamran University of Ahvaz, Ahvaz, IR Iran; 2 Department of Food Hygiene, Faculty of Veterinary Medicine, Shiraz University of Medical Sciences, Shiraz, IR Iran

**Keywords:** Silver, Nanoparticles, Pathogen, Disinfection, Microbial Sensitivity Tests

## Abstract

**Background:**

There is increased demand for improved disinfection methods due to microorganisms resistant to multiple antimicrobial agents. Numerous types of disinfectants are available with different properties; but the proper disinfectant must be carefully selected for any specific application to obtain the desired antimicrobial effect.

**Objectives:**

Antimicrobial effect of a commercial nanosilver product, NanoCid® L2000, against some foodborne pathogens was evaluated.

**Materials and Methods:**

Minimum inhibitory concentrations (MIC) were determined by monitoring the growth of bacteria at 600 nm, after 24 hours incubation at 35°C. Minimum bactericidal concentrations (MBC) were determined based on 3 log decrease in the viable population of the pathogens after incubation of nutrient agar plates at 35°C for 24 hours. The required exposure time for 3 log reduction in the viable population of the tested pathogens was determined as the minimum exposure time for efficient bactericidal activity.

**Results:**

The MIC values of Ag NPs against tested pathogens were in the range of 3.12-6.25 µg/mL. While *Listeria monocytogenes* showed the MIC value of 6.25 µg/mL, *Escherichia coli* O157:H7, *Salmonella typhimurium* and *Vibrio parahaemolyticus* all showed the MIC values of 3.12 µg/mL. However, all the pathogens showed the same MBC value of 6.25 µg/mL. To obtain an efficient bactericidal activity against *E. coli* O157:H7 and *S. typhimurium*, the exposure time should be at least ca. 6 hours., while this time was ca. 5 hours for *V. parahaemolyticus* and ca. 7 hours for *L. monocytogenes*.

**Conclusions:**

Silver nanoparticles showed great antibacterial effectiveness on four important foodborne pathogens. Therefore, Ag NPs could be a good alternative for cleaning and disinfection of equipment and surfaces in food-related environments.

## 1. Background

For many decades, foodborne diseases have been noticed as serious threats to public health all over the world. In foodborne pathogens studies, four major pathogens have emerged significantly important in terms of human health and disease. These include: *Escherichia coli* O157:H7, *Listeria monocytogenes*, *Salmonella typhimurium* and *Vibrio parahaemolyticus*. These organisms have frequently been associated with food products and linked to a number of human illness cases (1). *E. coli* O157: H7 is an important global cause of diarrhea, hemorrhagic colitis and hemolytic-uremic syndrome. The illness is often linked to the consumption of contaminated and undercooked ground beef as well as unpasteurized fruit juices (2, 3). 

*L. monocytogenes* has been implicated in foodborne outbreaks and subsequently isolated from various products such as meat, milk and milk products, vegetables, poultry, and fish (4). *Salmonella* is an important pathogen that causes major problems of morbidity and mortality around the world. Meat and poultry industries are the main reservoir of *Salmonella* as a foodborne pathogen. *S. typhimurium*has been the most common serotype associated with laboratory-confirmed illness cases (5, 6). *V. parahaemolyticus* is a human pathogen that occurs naturally in the marine environments and has been frequently isolated from a variety of seafood including fish, shrimp, crab, lobster, scallop and oyster (7).

The risk of foodborne diseases can be reduced by adopting some simple precautions such as avoiding cross contamination as well as employing good hygienic practices. One of the major causes of several outbreaks is believed to be lack of or insufficient cleaning and disinfection of equipment and surfaces in food-related environments. Therefore, many of foodborne diseases could be prevented by targeted disinfection in mentioned areas. Numerous types of disinfectants are available with different properties, the proper disinfectant must be selected carefully for the specific application to obtain the required antimicrobial effect (8-10). With the emergence of microorganisms, resistant to multiple antimicrobial agents, there is increased demand for improved disinfection methods. Therefore, new technologies have been used for efficient disinfection and microbial control.

During the past few decades, nanotechnology has emerged up as a new promising technology for synthesis of nanomaterials, particles in the nanometer size, which exhibit antimicrobial effects owing to their high surface-area–to-volume ratio and unique chemical and physical properties (11, 12). The bactericidal effects of various metallic nanoparticles including copper, titanium, zinc and silver, have been well documented (13). Silver has been known to have a disinfecting effect as well as applications in traditional medicine and culinary items (14). As early as 1000 B.C. (Before Christ), silver was used to make water potable (15). High antimicrobial efficacy of ionic silver (Ag^+^) against a broad spectrum of Gram positive and Gram negative bacteria as well as fungi (16) in combination of low toxicity against human tissue (17) has been led to the wide application of elemental silver or silver compounds in medicine. Hence, Silver nanoparticle (Ag NPs) is a good candidate as an alternative for formulation of a new generation of antibacterial agents used in biological, medical, and pharmaceutical applications (18-20).

## 2. Objectives

The present study was carried out to investigate the antimicrobial effect of a commercial nanosilver product, NanoCid® L2000, against representative microorganisms of public concern in food-related environments. The antimicrobial effect of silver nanoparticles was assessed by determining the minimum growth inhibitory concentrations (MIC) and minimum bactericidal concentration (MBC). In addition, the minimum exposure time for efficient bactericidal activity of this nanosilver product was determined.

## 3. Materials and Methods

### 3.1. Preparation of Nanosilver Solution

A stock solution of nanosilver with the average size of ca. (about) 10 nm was prepared from a liquid (L)-form of a nanosilver colloid product (NanoCid® L2000, Nano Nasb Pars Co., Tehran, Iran). The stock solution was then used to prepare the subsequent dilutions; 100, 50, 25, 12.5, 6.25, 3.12, 1.56 and 0.78 µg/mL, using serial two-fold dilutions.

### 3.2. Microorganisms

The stock cultures of *E. coli* O157:H7 (ATCC 43895), *L. monocytogenes* (ATCC 7644), *S. typhimurium* (ATCC 35987) and *V. parahaemolyticus* (ATCC 35118) were stored at -20°C in Tryptic soy broth (TSB) (Merck, Germany) supplemented with 25% (v/v) sterile glycerol (Merck, Germany). Test organisms were first activated by two successive transfers in TSB at 35°C for 24 hours.

### 3.3. Preparation of the Inoculums

100 µL of the overnight cultures of each bacterium were transferred to 10 mL TSB and incubated at 35°C with shaking. Absorbance of the cultures were measured at 600 nm after 5 hours and the viable cell count at this absorbance was determined by plating onto tryptic soy agar (TSA). According to the correlation between absorbance and viable cell count, approximately 105 - 106 cfu/mL of each bacterium was inoculated into the wells of the microplates.

### 3.4. Determination of MIC and MBC

MIC, defined as the lowest concentration of an antimicrobial agent that inhibits the growth of a microorganism after overnight incubation, was determined by monitoring the growth of bacteria in a microplate reader (Synergy HT, BioTek Instruments) at 600 nm. Serial two-fold dilutions of nanosilver solution were prepared in sterile 96-well plates over the range of 0.78-100 µg/mL. The wells were then inoculated with diluted overnight broth culture to give the initial population of 105 - 106 cfu/mL and incubated at 35°C for 24 hours. The bacterial growth was defined as absorbance increase at 600 nm with shaking for 30 seconds before reading. All the experiments were carried out at least six times.

MBC, the lowest concentration of nanoparticles that kills ≥ 99.9% (3 log) of the bacteria, was also determined. For this, samples were taken from the wells showing no growth, spread onto nutrient agar plates and incubated at 35°C for 24 hours. MBC was determined based on 3 log decrease in the viable population of the pathogens.

### 3.5. Determination of Minimum Exposure Time for Efficient Bactericidal Activity

To determine the minimum exposure time for efficient bactericidal activity of Ag NPs, the viable populations of all tested bacteria were determined during a period of 8 hours incubation in the absence (controls) or presence (treatments) of the relevant MBCs (6.25 µg/mL). The exposure time needed for ≥ 99.9% (nanosilver log) reduction in the viable population of the tested pathogens was defined as the minimum exposure time for efficient bactericidal activity.

## 4. Results

As shown in Table 1, the MIC values of Ag NPs against tested pathogens were in the range of 3.12-6.25 µg/mL. While *L. monocytogenes *showed the MIC value of 6.25 µg/mL, the three Gram negative tested pathogens showed the MIC values of 3.12 µg/mL. However, in our study all the pathogens showed the same MBC values of 6.25 µg/mL. 

**Table 1.** MIC and MBC Values of NanoCid® Against Foodborne Pathogens in Nutrient Broth

Bacteria	MIC	MBC
***Escherichia coli*** ** O157:H7**	3.12	6.25
***Listeria monocytogenes***	6.25	6.25
***Salmonella typhimurium***	3.12	6.25
***Vibrio parahaemolyticus***	3.12	6.25

**Figure 1.**
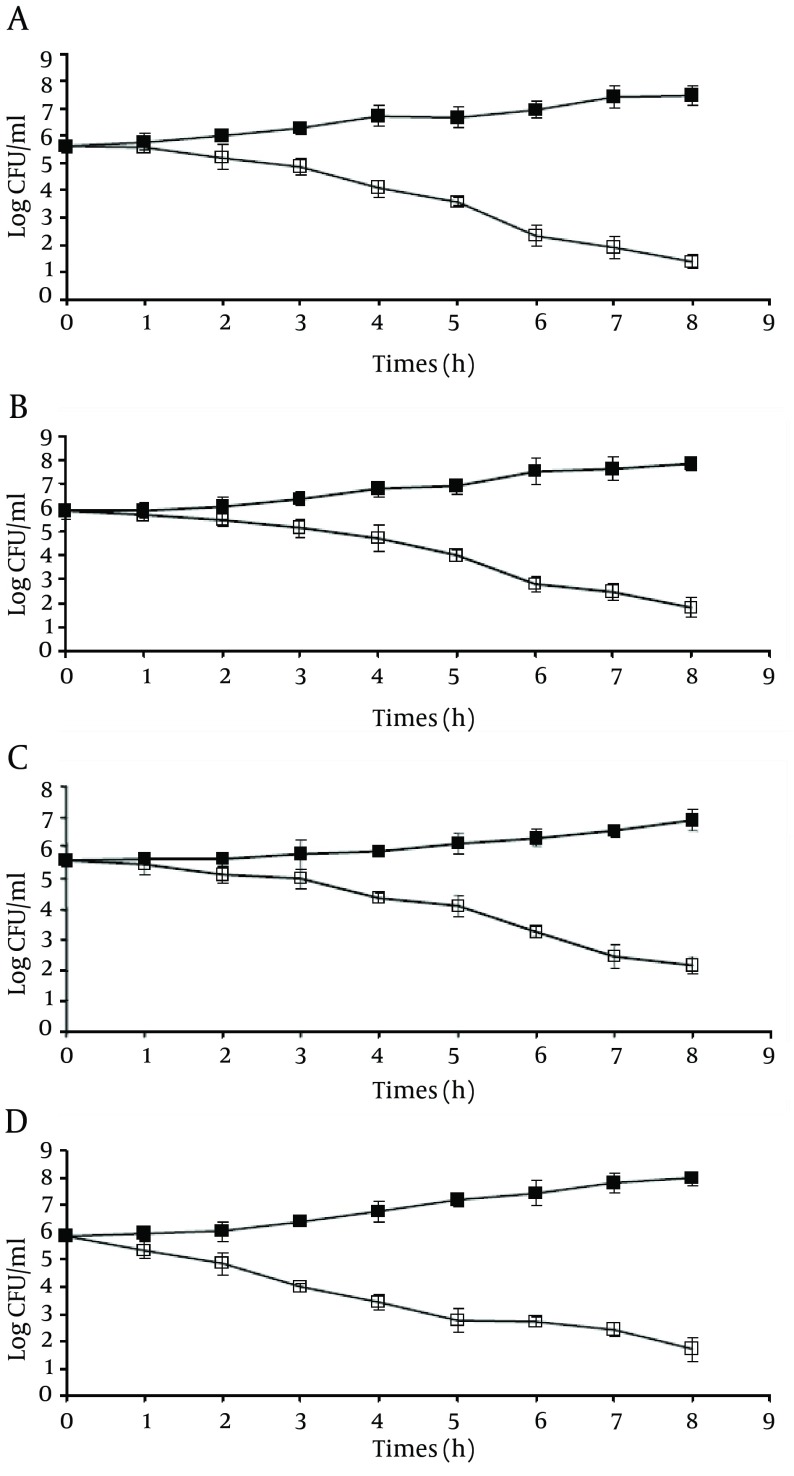
Growth and Survival Profile of the Assessed Bacteria A) E. coli O157:H7, B) S. typhimurium, C) L. monocytogenes, and D) V. parahaemolyticus in the absence (controls, ■) or presence (treatments, □) of 6.25 µg/mL of NanoCid®.

In an attempt to determine the minimum exposure time for efficient bactericidal activity of silver nanoparticles against tested pathogens, our results showed that for ≥ 99.9% (3 log) reduction in the viable population of *L. monocytogenes *, contact time should be at least ca. 7 hours, while this time was ca. 5 hours for *V. parahaemolyticus *and ca. 6 hours for *E. coli *O157:H7 and *S. typhimurium*. As shown in Figure 1, after ca. 6 hours of incubation, viable populations of *E. coli *O157:H7 and *S. typhimurium *decreased from 5.64 and 5.85 log cfu/mL to 2.32 and 2.78 log cfu/mL, respectively (Figure 1 A and B). However, viable population of *L. monocytogenes *decreased from 5.59 to 2.46 log cfu/mL after ca. 7 hours of incubation and viable population of *V. parahaemolyticus *decreased from 5.87 to 2.75 log CFU/mL after ca. 5 hours of incubation. 

## 5. Discussion

Antibacterial activity of silver nanoparticles has been demonstrated in several investigations, but the reported MIC values range through a wide extent of variation. Hence, it is difficult to compare their results, because there is no standard protocol for evaluation of antimicrobial activity of nanoparticles and different methods have been used by researchers. In the present study, silver nanoparticles showed good antibacterial activity against all the tested pathogens. The results of MIC and MBC tests revealed a higher MIC value for *L. monocytogenes* comparing to the other tested pathogens. This may be due to the differences in bacterial cell walls, since Gram negative bacteria have thinner cell wall comparing to Gram positive bacteria (21). 

In agreement, Kim et al. reported that *S. aureus* was more resistant against nanosilver than Gram negative *E. coli *(12). However, in our study, the MBC values were identical for all the pathogens. It has been previously stated that bactericidal property of nanoparticles is dependent on the concentration and size of nanoparticles and also the initial bacterial concentration (22). Silver nanoparticles with size of 1-10 nm have been reported to be most effective against bacteria through direct interaction with bacterial cells (11). For example, MIC was reported to be in the range of 3 - 25 µg/mL for *E. coli* at initial concentration of 105-108 cfu/mL and colloidal silver nanoparticles with the size range of 2 - 25 nm. Furthermore, Pal et al. found that interaction of nanoparticles with *E. coli *was shape-dependent, since truncated triangular particles showed higher activity compared to spherical and rod-spherical particles (23).

Since, in disinfecting the food-related environment, the exposure time for an efficient bactericidal activity is important, the minimum exposure time for ≥ 99.9% reduction in the viable population of the pathogens was also determined. The lowest and the highest exposure times were observed for *V. parahaemolyticus *and *L. monocytogenes *, respectively. As shown in Figure 1 A-D, in the presence of 6.25 µg/mL silver nanoparticles (relevant MBCs), viable population of all the pathogens decreased as the incubation time increased. However, our results showed that to obtain an efficient bactericidal activity against *E. coli *O157:H7 and *S. typhimurium *, the exposure time should be at least ca. 6 hours, while this time was ca. 5 hours for *V. parahaemolyticus *and ca. 7 hours for *L. monocytogenes *. 

Silver nanoparticles showed great antibacterial effects on four important foodborne pathogens. Therefore, with development of multidrug-resistant strains of bacteria, Ag NPs could be good alternatives for cleaning and disinfection of equipment and surfaces in food-related environments.
